# Mixed-Matrix Organo–Silica–Hydrotalcite Membrane for CO_2_ Separation Part 2: Permeation and Selectivity Study

**DOI:** 10.3390/membranes14070156

**Published:** 2024-07-12

**Authors:** Lucas Bünger, Tim Kurtz, Krassimir Garbev, Peter Stemmermann, Dieter Stapf

**Affiliations:** Institute for Technical Chemistry, Karlsruhe Institute of Technology, Kaiserstrasse 12, 76131 Karlsruhe, Germany; tim.kurtz@kit.edu (T.K.); krassimir.garbev@kit.edu (K.G.); peter.stemmermann@kit.edu (P.S.); dieter.stapf@kit.edu (D.S.)

**Keywords:** inorganic membrane, silica membrane, hydrotalcite membrane, CO_2_/N_2_ separation, microporous mixed-matrix membrane

## Abstract

This study introduces an innovative approach to designing membranes capable of separating CO_2_ from industrial gas streams at higher temperatures. The novel membrane design seeks to leverage a well-researched, high-temperature CO_2_ adsorbent, hydrotalcite, by transforming it into a membrane. This was achieved by combining it with an amorphous organo-silica-based matrix, extending the polymer-based mixed-matrix membrane concept to inorganic compounds. Following the membrane material preparation and investigation of the individual membrane in Part 1 of this study, we examine its permeation and selectivity here. The pure 200 nm thick hydrotalcite membrane exhibits Knudsen behavior due to large intercrystalline pores. In contrast, the organo-silica membrane demonstrates an ideal selectivity of 13.5 and permeance for CO_2_ of 1.3 × 10^−7^ mol m^−2^ s^−1^ Pa^−1^ at 25 °C, and at 150 °C, the selectivity is reduced to 4.3. Combining both components results in a hybrid microstructure, featuring selective surface diffusion in the microporous regions and unselective Knudsen diffusion in the mesoporous regions. Further attempts to bridge both components to form a purely microporous microstructure are outlined.

## 1. Introduction

Providing captured CO_2_ from industrial exhaust streams is a basic premise for circular processing e.g., in methanization or reverse water–gas shift. A continuous and concentrated CO_2_ stream at high temperatures is necessary for efficient process operation [1]. However, no separation process currently operates continuously without experiencing a temperature drop [2].

**Figure 1:**
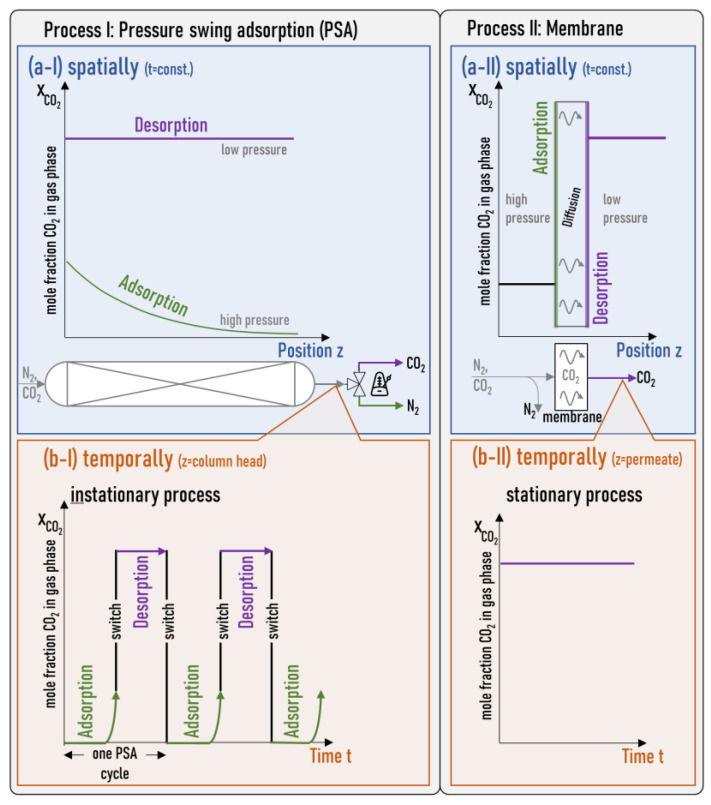
Conceptual comparison of PSA and membrane processes for CO_2_/N_2_ separation. Panels (**a**) show the CO_2_ mole fraction X_CO2_ in the gas phase as a function of the position z: (**a-I**) in the adsorption column and (**a-II**) in the membrane at a point in time t. Panels (**b**) show the CO_2_ mole fraction in the gas phase as a function of the time: (**b-I**) at the column head for the PSA and (**b-II**) at the permeate side for the membrane. In the process I (PSA), adsorption and desorption take place at the same location (**a-I**) but at different times (**b-I**). In process II (membrane), adsorption and desorption are spatially separated (**a-II**) but occur simultaneously (**b-II**).

State-of-the-art separation processes for carbon capture, such as methanol scrubbing, operate at low temperatures, while pressure-swing adsorption (PSA) yields CO_2_ pseudo-continuously [3]. Current developments facilitate adsorption at higher temperatures by employing appropriate high-temperature adsorbents and implementing intelligent process interconnections. However, this approach requires relatively high operating costs and capital investment. Membrane processes might offer a solution by utilizing separation layers that incorporate adsorbents, allowing for the separation of CO_2_ at elevated temperatures [4]. These processes can provide continuous volume flows by spatially separating adsorption and desorption through diffusion across the membrane (see Figure 1). Therefore, membrane processes can be viewed as spatially separated PSA applications.

Gas-separation membranes, both pure polymeric and mixed-matrix membranes with inorganic fillers, show great CO_2_/N_2_ separation quality; however, they fail to maintain that behavior at high temperatures [5,6]. This work tries to offer a new route in developing high-temperature gas-separation membranes by combining high-temperature adsorbents within well-known inorganic xerogel matrices. Therefore, the focus is on the design process and the general feasibility of the preliminary membrane.

To design selective membranes, the mass transport mechanisms of the respective gas components in the porous membrane comprised the critical design criteria. The mass transport characteristics in porous materials are primarily influenced by the size of the pore diameter, d_p_. In macro pores (dp > 50 nm), the primary mechanism is viscous flow. Smaller pores within the mesopore range (2 nm < dp < 50 nm) experience an increased prominence of Knudsen diffusion. Mass transport kinetics in these pores are influenced by the molecular weight. In membranes containing micropores (dp < 2 nm), the impact of the internal surface becomes relevant, facilitating the adsorption/desorption behavior of gas components as an additional mass transport effect. In such cases, the membrane material becomes the dominant factor influencing mass transport kinetics, given the significance of mass transport over the surface. Within the ultramicroporous range (dp << 1 nm), the selective exclusion of gaseous molecules begins, resulting in a decline in permeation or molecules larger than the pore opening, eventually approximating zero. Membranes featuring pores within the molecular sieving range can demonstrate significant selectivities [7,8]. Nevertheless, such membranes often display very low fluxes for the permeating component relative to membranes with larger pores [9]. The same low-flux phenomena are observed for molten hydroxides and carbonate membranes operating at high temperatures [10], only showing good permeances above 650 °C–850 °C [11]. For efficient membrane design, only membranes with pore sizes below 2 nm, made of a CO_2_ adsorption agent, are the most efficient solution for CO_2_ gas separation [7,8].

Several researched membrane materials exhibit microporosity. Various zeolites [12], metal–organic frameworks (MOFs) [13], carbon-based membranes [14], and sol–gel-derived silica gels [15,16] are suitable materials for this separation challenge. In this study, silica membranes were chosen due to their relatively simple sol–gel coating production process and their ability to achieve CO_2_ selectivity while maintaining adequate permeability.

**Figure 2:**
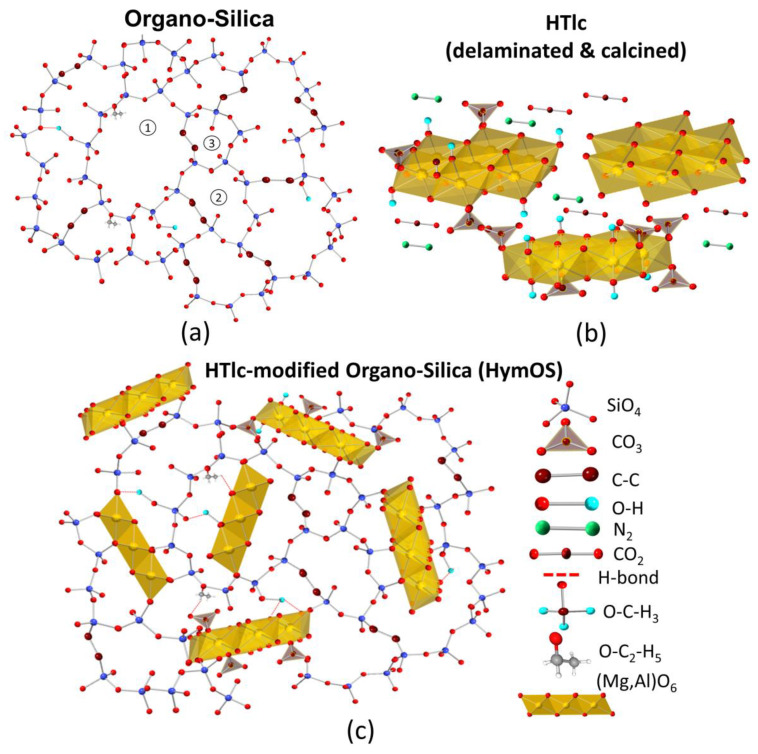
Schematic visualization of the microstructures of (**a**) organo–silica (pores with different sizes and resulting mass transport mechanism are marked by (1), (2) and (3), (**b**) the pure calcined hydrotalcite, (**c**) and the hydrotalcite-modified organo–silica (HymOS) as the combination of both (**c**).

Figure 2a portrays the inorganic xerogel organo–silica structure, wherein mass transport occurs along the interior of the silica network through pores of different sizes (pores with different sizes marked by (1), (2), and (3)). The selectivity of silica membranes is primarily based on their adsorptive affinity for CO_2_. However, the utilization of silica membranes is limited to lower temperature ranges (<200 °C) due to a strong decrease in their adsorption capacity upon increasing temperatures [17,18]. For a membrane to effectively separate CO_2_ at high temperatures, the active membrane material needs to have adsorption sites with an exothermic heat of adsorption at the operating temperature [19]. Strong interaction of adsorption sites can hinder the mobility of the adsorbed gaseous component, leading to reduced diffusion [20]. Therefore, it is crucial to find the right adsorption agent.

The field of high-temperature adsorbents has received a lot of attention, leading to the identification of layered double hydroxides (LDH) as a suitable precursor for CO_2_ adsorption applications at elevated temperatures [21,22,23,24]. The used LDH can differ in the composition of cations (e.g., Mg, Al, Fe, Co, Mo, etc.), anions (e.g., sulfate, chlorides, and nitrates), structural order (crystalline, partially disordered, or amorphous), as well as in content and bonding of hydrogen (hydrous/anhydrous, partially dehydroxylated up to hydrogen-free, or “mixed oxides”) [25]. While there is extensive research on the use of calcined hydrotalcite-like components (HTlcs) as an adsorbent, there have been relatively few investigations regarding its application as an active separation agent, particularly as the sole membrane material for gas separation. Membranes solely made of LDHs yielded low selectivity for CO_2_, as the fabricated membranes showed intercrystalline pore structures with average diameters of permeation that favored Knudsen diffusion [26,27]. Figure 2b shows the delaminated calcined HTlc membrane microstructure schematically as a result of thermal treatment and delamination. The permeation of the gases is through the volume that is opened by the effective average diameter between the HTlc sheets.

The approach to overcome the selectivity problem is demonstrated by mixing a crystalline inorganic compound with high selective surface diffusion properties into an amorphous matrix, as shown in Figure 2c. This approach has already been demonstrated for polymeric matrices in a wide range of combinations with different fillers [28]. There are several approaches for the use of LDH as a filler in polymeric matrices forming classic mixed-matrix membranes (MMM). They all show good separation behavior but have limited temperature stability and low permeance [29]. However, MMMs with matrices made of inorganic xerogels are not well investigated yet. Embedding a sorption-enhancing agent into a silica membrane was studied by the group of Othman and Wiheeb with tetraethylorthosilicate as a precursor for silica, which is very sensitive to moisture for applications in flue gases with water content [30,31,32,33,34,35].

This work presents a possible way to combine the adsorptive effect of calcined HTlc with an amorphous silica matrix to enhance the separation performance at high temperatures. First, a description of the individual preparation methods for silica and hydrotalcite membranes as well as for the combination of the two materials is given. Second, the experimental setup for conducting mass transport experiments with the prepared membrane is illustrated. Finally, the preparation techniques and the corresponding mass transport performance are evaluated.

## 2. Materials and Methods

### 2.1. Membrane Fabrication

As a substrate for membrane deposition, a macroporous α-alumina disc with a thickness of 2.2 mm and a diameter of 39 mm and an approximate pore size of 80 nm of its top layer (Pervatech, The Netherlands) was used. On the top of the α-Al_2_O_3_ substrate, a supportive intermediate mesoporous γ-Al_2_O_3_ layer was deposited, produced by sol–gel synthesis, using a thermally treated boehmite (γ-AlO(OH)) sol after the method of Yoldas [36]. To obtain reproducible layer thickness, 3.5 g of polyvinylalcohol (PVA) was dissolved in 100 mL water and mixed with the boehmite sol in a ratio of 2:3.

A detailed description of the mass transport-enhancing compound synthesis, hydrotalcite, and respective analysis of all steps are given in Part 1 of this work.

The HTlc used in this work was synthesized by dissolving 6.1 g of magnesium chloride hexahydrate and 2.41 g of aluminum chloride hexahydrate, with a molar ratio of Mg/Al = 3, in 100 mL methanol. This mixture was heated to 65 °C and stirred under reflux. The pH was set by a solution of 3.8 g of sodium hydroxide in 100 mL of methanol that was added dropwise to the metal–salt solution [37,38]. For a small particle size distribution, the synthesis time was reduced from 3 days to 1 h.

After cooling the reaction mixture to room temperature, it was centrifuged, and the remaining pellet was dispersed in water. This process was repeated until the pH became neutral. The remaining pellet was then dispersed in water and left overnight to delaminate. As a result, a clear and stable dispersion of delaminated hydrotalcite-like nanosheets was produced. This sol was used to fabricate the pure HTlc membrane. After coating and drying, thermal treatment to activate the agent was carried out at 400 °C for 3 h in air [21].

The synthesis of the microporous organo–silica membrane (Org-Sil) for CO_2_ separation was performed after the approach of van Gestel et al. and Castricum et al. [18,39]. It involved mixing 16.655 mL of the membrane-forming precursor, 1,2-bis(triethoxysilyl)-ethane (BTESE), with 28.14 mL of ethanol (both Thermo Fisher (Kandel) GmbH, Kandel, Germany), 0.63 mL of nitric acid (65 wt%), and 4.57 mL of water. These amounts result in a water-to-hydrolysable ethoxy group ratio of one. The resulting mixture was heated to 60 °C and refluxed for 90 min, followed by cooling to room temperature. The sol was stabilized for storage by the addition of 50 mL ethanol. The resulting mixture was stored in the refrigerator (stock sol). For the coating process in terms of the formation of defect-free films, the mixture was diluted in a ratio of 1 to 20 with ethanol. After coating and drying at room temperature for 1 h, thermal treatment of the membrane was conducted under a N_2_ atmosphere at 300 °C for 3 h [39,40].

A detailed description of the process to transfer the water-based HTlc sol into a mixable solution with silica is given in Part 1 of this study. The stable delaminated HTlc sol was first dried and then calcined at 400 °C. Two grams of the resulting calcined and ground hydrotalcite were redispersed in ethanol with the assistance of an ultrasonic device.

To ensure the removal of undispersed particles, the dispersion was subjected to centrifugation for 20 min at 4000 rpm, resulting in a stable dispersion of calcined delaminated hydrotalcite-like nanosheets.

Various mixing ratios of both sols were explored, but only the lower and higher ends of the mixing range are presented here.

At the lower end of the mixing range, 100 mL of the same silica sol used for the pure organo–silica (Org-Sil) membrane was combined with 5 mL of ethanol-based hydrotalcite sol to form the HymOS1-sol. At the higher end of the mixing range, 100 mL of the hydrotalcite dispersion was mixed with 5 mL of the silica stock sol, resulting in the HymOS2-sol. After 30 min of mixing, the mixtures were ready for coating onto the substrate to form the mixed-matrix membranes. The resulting as-deposited film was then allowed to air dry for approximately 1 h. Subsequently, thermal treatment was carried out at 300 °C for 3 h under a N_2_ atmosphere.

The coating process for all the aforementioned sols was performed uniformly. A 39 mm disc, used as the substrate, was attached to a vacuum suction cup located at the end of a rotating pendulum. A petri dish containing 50 mL of the respective sol was positioned at the lowest point of the pendulum. The coating process involved immersing the polished surface of the substrate into the sol with a controlled and defined velocity.

Once the pendulum reached a perpendicular position, its movement was halted for 15 s. After this pause, the substrate was rotated out of the sol with the same velocity as before. To prevent cracking caused by thermally induced tension, both the heating and cooling rates during thermal treatment were controlled to be 1 K/min. To ensure the production of crack-free membranes, all coating and thermal treatment processes were conducted twice. Afterward, the membranes were ready for structural and permeation analysis.

### 2.2. Structural Characterization

A high-resolution scanning electron microscope (SEM) was used for qualitative assessment of the prepared membrane layers. Images were captured using the Zeiss Supra 55 VP microscope with an acceleration voltage of 18 kV.

To prepare the membrane for SEM analysis, a crucial step involved freezing the silica layer by placing the membrane disc in liquid N_2_. This step was essential for obtaining high-quality images, as attempts to analyze the membrane without this freezing step resulted in images with varying quality. After spending a few minutes in liquid N_2_, the membrane was cut into small 1 mm^2^ pieces using a side cutter. In a further step, the pieces were then sputtered with gold to ensure electrical conductivity.

Additional analyses by X-ray diffraction, IR, and Raman spectroscopy and thermal analysis of the products after every step of preparation are extensively described in Part 1 of this study.

### 2.3. Gas Permeation and Separation Experiments

A custom-made cell designed to hold 39 mm membrane discs was used for single-gas experiments. The top of the membrane was sealed via a 32 mm Viton O-ring to the cell, resulting in an effective permeation area (A_m_) of 804 mm^2^. The thermal stability of the Viton limited the use of this setup to temperatures up to 200 °C.

The gases used in this study were delivered from gas bottles with reducers, allowing measurements at pressures up to 6 bar. They were fed into the cell via an inlet on the top side. The setup also included a retentate outlet (top side) as well as a permeate outlet at the bottom side of the cell. A Bronkhorst El Press pressure controller valve was installed in the retentate line. Pressure measurements were performed in the feed (p_f_) and the permeate (p_p_) line.

The mass flow of the permeating stream was quantified by three Bronkhorst EL-Flow Prestige mass flow meters with different measurement ranges (0–1 g/h, 0–10 g/h, and 200 g/h) to reduce the measurement error (error: ±0.5% of the measured value; ±0.1% of endpoint value). A membrane pump (KNF LABORPORT N938.50) installed in the permeate line allowed vacuums as low as 70 mbar. To measure the membrane performance at different temperatures, the module was placed in an oven. The temperature was controlled with a thermocouple installed in the proximity of the membrane. Each measurement was taken in steady-state for above an hour. For each membrane type, multiple membranes were tested. However, only a representative single membrane was used for the displayed results to reduce the impact of cracks and voids.

The permeance (Q_i_) for a gaseous component i is calculated from the measured membrane area, transmembrane pressure difference, and mole flow J_i_, according to Equation (1):

(1) $$Q_{i}=\frac{J_{i}}{A_{m}\cdot (p_{i,f}-p_{i,p})}=\frac{P_{i}}{d_{layer}}$$

The ideal selectivity S_CO_2_/N_2__ as the ratio of the single-gas permeances is calculated by Equation (2):

(2) $$S_{CO_{2}/N_{2}\;}=\frac{Q_{CO_{2}}}{Q_{N_{2}}}$$

A resistance-in-series model was utilized to determine the successive mass transport resistances in the composite membrane [26]. The resistance of each layer is inversely proportional to the intrinsic mass transport kinetics of the respective layer, expressed by its permeability P_i,layer_. Sequential measurements of each membrane layer provide the permeance (Equation (1)), and using the corresponding membrane thickness (d_layer_) obtained from SEM images, the permeability is calculated according to Equation (3).

(3) $$Q_{i,composite}={\left(\frac{d_{\alpha -Al_{2}O_{3}\;}}{P_{i,\alpha -Al_{2}O_{3}}}+\frac{d_{\gamma -Al_{2}O_{3}\;}}{P_{i,\gamma -Al_{2}O_{3}}}+\frac{d_{org-sil\;}}{P_{i,org-sil}}\right)}^{-1}$$

## 3. Results

### 3.1. HTlc Membrane

**Figure 3:**
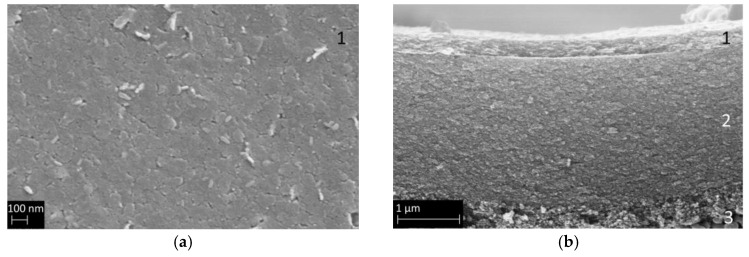
SEM secondary electron images of (**a**) the surface of calcined HTlc membrane (1) and (**b**) a cross-section of the composite membrane with 200 nm calcined HTlc on top of the mesoporous γ-Al_2_O_3_ mesolayer (2) and the pervatech macroporous support (3).

Figure 3 presents SEM images of a pure calcined delaminated hydrotalcite-like membrane, showing the surface (a) and the cross-section (b). Panel a shows that all crystals are preferentially oriented parallel to the substrate, which is a result of the membrane-coating process. Figure 3b shows a defect-free calcined HTlc layer with a thickness of 200 nm on top of a 2.2 µm thick γ-Al_2_O_3_ layer.

**Figure 4:**
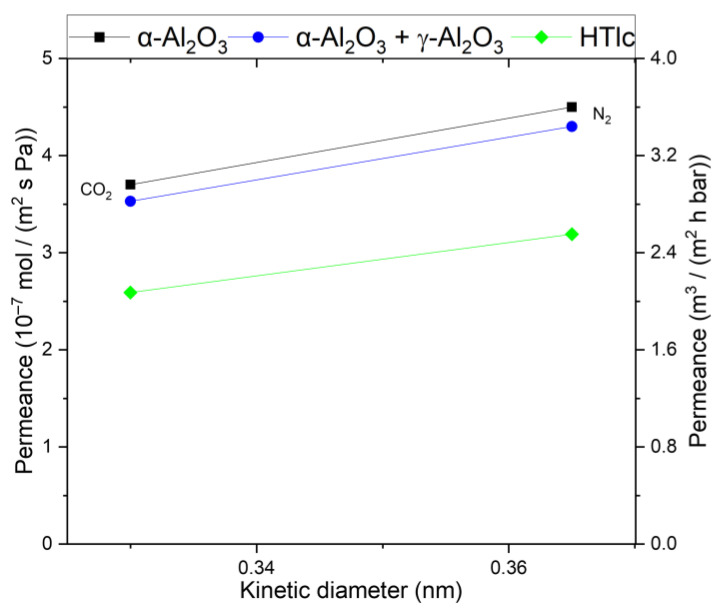
Single-gas permeation through the α-Al_2_O_3_ support (squares), the support + γ-Al_2_O_3_ (circle), and the pure hydrotalcite membrane on top of both (diamond) at 25 °C and 3.2 bar. Permeances for both CO_2_ and N_2_ are in scientific and technical units.

For a better comparison and understanding of the influence of the support layers of the membrane, a consecutive investigation of the membrane layers was conducted. Figure 4 shows the permeance of the substrate and the substrate + γ-Al_2_O_3_ layer, respectively. The supporting layers show Knudsen behavior; the pure support shows slightly higher permeance than the combination of the support with the γ-Al_2_O_3_ layer. This is as expected since the γ-Al_2_O_3_ layer introduces an additional resistance.

The permeance in the HTlc membrane is lower for both components (2.5 × 10^−7^ mol m^−2^ s^−1^ Pa^−1^ for CO_2_ and 3.1 × 10^−7^ mol m^−2^ s^−1^ Pa^−1^ for N_2_) than in the supporting layers, demonstrating a further resistance for the permeating gas molecules. Additionally, N_2_ is the primary permeating component, displaying a similar behavior to γ-Al_2_O_3_ and the substrate, albeit at a lower level. This suggests that the pure calcined HTlc layer possesses a structure with fewer or smaller pores than the other layers. The selectivity for CO_2_ at 20 °C is 0.81, indicating that the diffusion through this layer is primarily governed by Knudsen diffusion. Based on the selectivity, the size of the pores is in the same range as in the support. However, the lower flux is a consequence of the orientation of the crystals. They are arranged parallel to the substrate surface, thereby blocking a portion of the flow, as there is no direct path for diffusion through the crystal.

To our knowledge, no pure hydrotalcite membrane with surface diffusion as the predominant mass transport mechanism has been reported yet. However, its theoretical feasibility is convincing. For a material to have surface diffusion that can impact the overall mass transport its heat of adsorption is crucial. Calcined hydrotalcite exhibits a heat of adsorption for CO_2_ in the range of 40–50 kJ mol^−1^ at temperatures between 573 K and 623 K [41,42]. This enables surface diffusion according to the correlation presented by Yang [20]. The resulting surface diffusion coefficient ranges in the range of 10^−4^ to 10^−5^ cm^2^s^−1^. This is in the same range as the diffusion of CO_2_ on silica at 300 K [43]. This shows that calcined HTlcs are a suitable material for enhancing mass transport at elevated temperatures.

However, the way the crystals are arranged always leads to inter-crystalline pores in the range where Knudsen diffusion is dominating (see Figure 2b). Therefore, it is not possible to take advantage of the CO_2_ affine surface. Several studies have observed the similar diffusion through pure hydrotalcite layers with permeabilities in the same range (see Table 1) but always with selectivities in the Knudsen range [26,27]. To shift the mass transport from diffusion through the pore volume to surface diffusion, the pore volume must be converted into a microporous structure. Approaches to achieve this include stacking the hydrotalcite layers up to 300 µm thick packages that show selectivity for CO_2_ but lead to very low permeances due to the enhanced layer thickness [26]. More promising is the effort to fill the pore volume with a permeable material to reduce the effective average diameter of the permeation path that still allows for economically feasible flows. This leads to mixed-matrix membranes that utilize polymers as the permeable matrix. Tsotsis et al. tried silicone as a matrix material with success, creating membranes with high selectivities that were, however, still on a low permeance level and with no application at higher temperatures [27].

The novel approach in this work focuses on exchanging the polymer with a temperature-stable inorganic material (see Figure 2c). The challenge lies in creating a microporous microstructure where an inorganic organo–silica matrix fills up the free space between the HTlc phase. This tries to transfer the idea of organic matrices with an inorganic filler to a membrane where the matrix is made of an inorganic material, too.

### 3.2. Pure Organo–Silica Membrane (Org-Sil)

**Figure 5:**
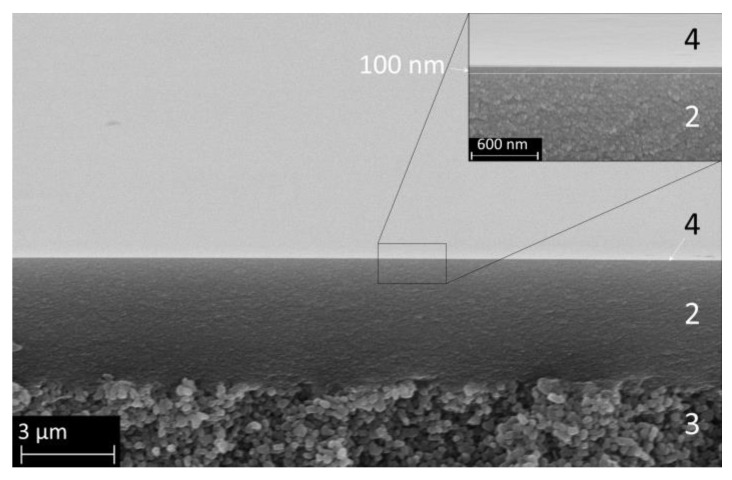
SEM image of the microporous organo–silica top layer (4) on top of the γ-Al_2_O_3_ layer (2) and the α-Al_2_O_3_ support (3). The insert shows the same membrane at a higher magnification for better visualization of the organo–silica layer thickness.

To understand the inorganic matrix material, its permeating properties as a single layer were investigated. Figure 5 displays SEM images of an organo–silica membrane in a cross-section with the surface visible in the upper part. Three distinct layers are visible. In the insert, the organo–silica top layer 4 is shown, with a thickness of about 100 nm and a defect-free, smooth surface.

**Figure 6:**
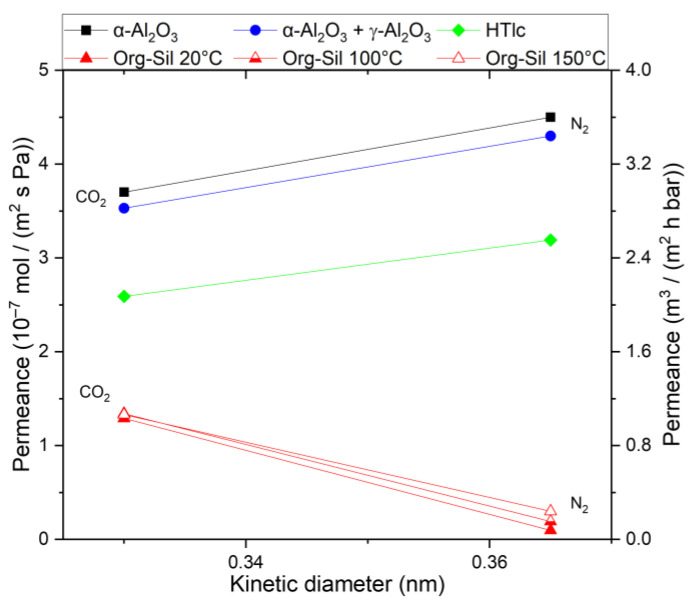
Single-gas permeation of CO_2_ and N_2_ through an Org-Sil membrane (red) at different temperatures, in reference to the permeances shown in Figure 2 at 25 °C. The transmembrane pressure was kept constant at 3.2 bar. Permeances for both CO_2_ and N_2_ are in scientific and technical units.

Single-gas permeation experiments were conducted with CO_2_ and N_2_ to determine the permeation kinetics (see Figure 6). The organo–silica layer deposited on the top of the γ-Al_2_O_3_ layer shows different permeation behavior compared to the other membranes. While both gases traverse micropores, surface diffusion emerges as the predominant mass transport mechanism. N_2_ can solely diffuse through the free pore volume because its adsorptive interactions are neglectable. The permeance decreases to approximately ~0.1 × 10^−7^ mol m^−2^ s^−1^ Pa^−1^ at room temperature. CO_2_, having an additional diffusion path along the inner pore surface, undergoes a smaller decrease, resulting in a permeance of around ~1.3 × 10^−7^ mol m^−2^ s^−1^ Pa^−1^. Consequently, the ideal selectivity reaches 13.

As temperature rises during organo–silica measurements, two distinct behaviors become apparent. Firstly, the permeance for CO_2_ appears to remain unaffected. As temperature increases, activated permeation rises, while surface diffusion, which relies on active adsorption sites, decreases [18]. These two mass transport mechanisms seem to counterbalance each other for this pore size in the case of CO_2_, resulting in a relatively consistent permeance as temperature rises. Secondly, the permeance for N_2_ increases. With increasing temperature, more of the required activation energy is provided for the activated permeation in ultramicropores. Surface diffusion does not participate in the overall N_2_ transport; thus, no observable decrease, as in the case of CO_2_, counters the increase. A comprehensive description of activated permeation can be found in the study conducted by Lange et al. [19]. Overall, because of the increasing N_2_ permeance, the selectivity diminishes to 4.3 at 150 °C.

While an efficient separation of N_2_ and CO_2_ is achievable at low temperatures, a decrease in separation efficiency at elevated temperatures is observed. This could be explained by the fact that whereas the CO_2_ permeance remains relatively constant at higher temperatures, the N_2_ permeance increases. The combination of organo–silica and HTlcs is expected to overcome the respective limitations in a novel MMM.

### 3.3. HTlc Modified Organo–Silica Membrane (HymOS)

**Figure 7:**
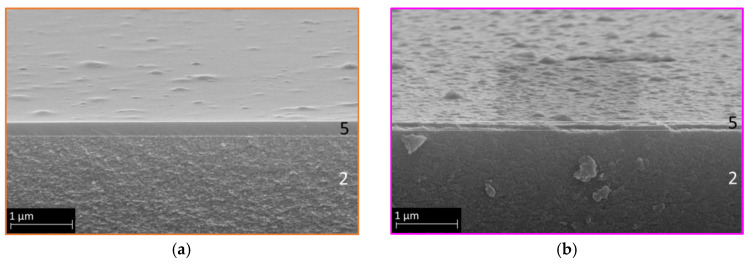
SEM secondary electron images of the HymOS1 membrane (5) on top of the γ-Al_2_O_3_ layer (2) (**a**) with a low hydrotalcite concentration and (**b**) the HymOS2 membrane with a higher concentration.

Figure 7 displays two SEM images of the modified membrane with different HTlc concentrations: lower concentration (a) and higher concentration (b). The preparation of mixed membranes proved to be challenging in terms of HTlc/sol ratio. Too little hydrotalcite in the silica sol would not notably alter the mass transport behavior, while an excess would enlarge the pores, creating voids and thus enhancing Knudsen diffusion, as seen in Figure 2c [32,44].

**Figure 8:**
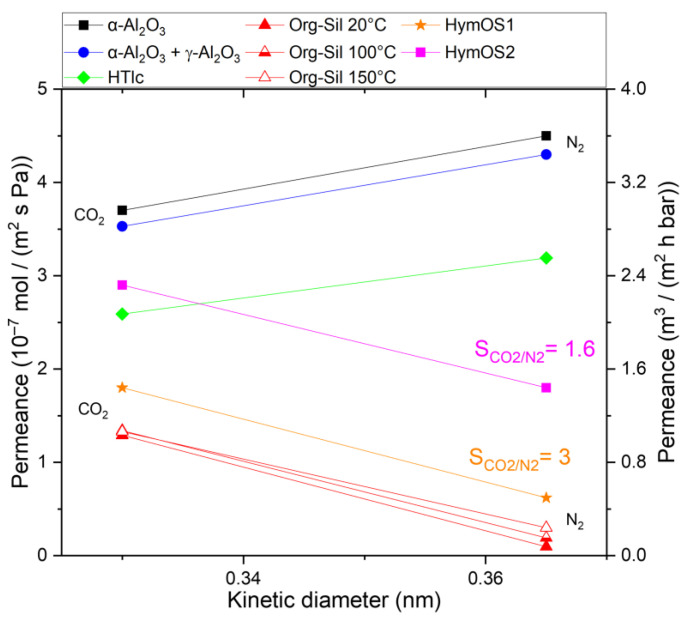
Single-gas permeation at 20 °C (other temperatures are indicated) through two modified membranes (HymOS1, orange star; HymOS2, pink square) in relation to the permeances of the single-component membranes described before. The transmembrane pressure was kept constant at 3.2 bar. Permeances for both CO_2_ and N_2_ are in scientific and technical units.

Both SEM images exhibit continuous and defect-free membranes with clearly seen differences in the hydrotalcite content. To assess the functionality of the prepared membranes, single-gas experiments were conducted. Figure 8 illustrates the permeance of HymOS1 and HymOS2. For a better comparison with other membranes. Table 1 lists the calculated (Equation (3)) single-layer, stand-alone permeabilities for all manufactured membranes in this work.

Both membranes exhibit higher permeance values for both components compared to the pure organo–silica membrane. This outcome aligns with expectations, as the presence of HTlcs disrupts the silica microstructure, increasing porosity and pore size. For HyMOS1, with lower hydrotalcite content, the permeance increased from around 1.4 × 10^−7^ mol m^−2^ s^−1^ Pa^−1^ to 1.9 × 10^−7^ mol m^−2^ s^−1^ Pa^−1^ for CO_2_ and from 0.1 × 10^−7^ mol m^−2^ s^−1^ Pa^−1^ to 0.6 × 10^−7^ mol m^−2^ s^−1^ Pa^−1^ for N_2_. Consequently, the selectivity dropped from 13 to 3. The second membrane (HymOS2), with higher hydrotalcite content, demonstrates similarly elevated permeance for both components, albeit at a higher level. With more hydrotalcite crystals further disrupting the microstructure, the ratio favoring Knudsen diffusion is intensified, leading to a further reduced selectivity of 1.6. The results indicate that the expected improvement in mass transfer along the HTlc surface was not observed. The increased Knudsen diffusion, caused by the disrupted microstructure, overrides the effect of additional surface diffusion. For membranes with a Knudsen diffusion microstructure, temperature-dependent mass transfer experiments were not conducted because Knudsen diffusion does not exhibit temperature-dependent selectivity.

## 4. Discussion

Research efforts have yielded various materials that potentially facilitate the selective separation of N_2_ from CO_2_. This study focused on utilizing a hydrotalcite-derived component in a way that has not been investigated yet. Numerous studies prepared membranes by use of some LDH-derived components [45]. Most are designed to facilitate selectivity by molecular sieving through the interlayer gallery. Some have prepared LDHs with CO_2_ transport channels that have some sort of carrier mechanic that is highly selective for CO_2_ [46]. Former membranes lack the applicability for flue gas cleaning, as the permeances are low and expected to decrease further if mixed-gas permeances were investigated. The transport channel membranes, however, show enormous permeances while having high selectivities. To our knowledge, the transport mechanism is highly pressure-dependent and reduces at technical operation points.

Therefore, this study attempted to introduce a membrane with a third option of facilitating high flux and high selectivity at elevated temperatures by utilizing calcined hydrotalcite, which has proven its application in high-temperature CO_2_-adsorbent processes. Creating selective CO_2_ diffusion paths by providing high-temperature adsorption sites within a matrix is a membrane design concept with little research yet.

For the insertion of an active separation agent into a permeable matrix to form a functioning membrane, an imagined resistance interconnection needs to be considered [47]. As proposed by Zimmermann et al., MMMs with polymeric matrices can be viewed as parallel resistance of the respective permeating components [48]. This approach is transferred to inorganic–inorganic hybrid microstructures. Equation (4) shows the effective permeance Q for CO_2_ and Equation (5) for N_2_ through an imagined MMM with the volume fraction Φ of the calcined HTlc. The permeance through the silica matrix is described by ($Q_{i,org-sil}$), exhibiting a decreasing portion of the overall permeance with increasing volume fraction of the HTlc phase.

(4) $$Q_{CO_{2},\;MMM}={(1-\phi_{HTlc})Q}_{CO_{2},\;org-sil}+\phi_{HTlc}\;(Q_{CO_{2},HTlc,S}+Q_{CO_{2},HTlc,Kn})$$

(5) $$Q_{N_{2},\;MMM}={(1-\phi_{HTlc})Q}_{N_{2},\;org-sil}+\phi_{HTlc}\;(Q_{N_{2},HTlc,S}+Q_{N_{2},HTlc,Kn})$$

**Figure 9:**
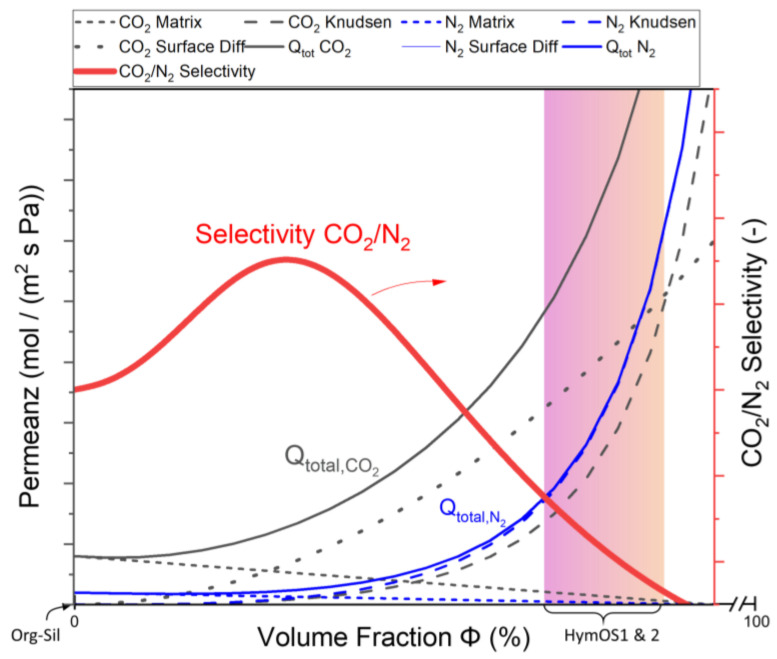
Visualization of the total MMM permeance described in Equation (2) for CO_2_ (black) and Equation (3) for N_2_ (blue) as a function of the added HTlc content (only the part for low volume fractions is shown here). The total permeance and the permeances for different mass transport mechanisms that comprise the total permeance are given in dark for CO_2_ and blue for N_2_. The resulting selectivity is shown in red. The prepared membranes HymOS1 and 2 lie within in the marked area.

For the mixture to be a selective membrane, the mass transport of CO_2_ along the surface of the HTlc ($Q_{CO2,HTlc,S}$) needs to reach higher values than for N_2_ ($Q_{N2,HTlc,S}$) at operating temperatures. Increasing the volume fraction of HTlcs improves the overall surface for CO_2_ diffusion. However, at the same time, the filler material causes the creation of voids that favor Knudsen diffusion ($Q_{i,HTlc,Kn}$), thus rendering the membrane less selective. Therefore, the membrane design needs to focus on finding the right amount of surface diffusion agent incorporated in the matrix to increase the selectivity. Figure 9 displays Equations (4) and (5) schematically to show the proposed design criteria. The Knudsen diffusion dominates the overall mass transport for high filler contents; therefore, Figure 9 only depicts low volume fractions and should be interpreted as an excerpt of the range from 0 to 100%.

For low HTlc content, CO_2_ surface diffusion increases more quickly than N_2_ Knudsen diffusion due to the additional free volume. In small pores, the added surface favors surface diffusion. This pattern reverses as the pores become larger because the volume of the pores increases more rapidly than the surface, leading to a preference for Knudsen diffusion with higher permeances. This results in an overall decrease in selectivity.

The prepared membranes in this work are located on the right side of Figure 9, indicated by the marked area. Both membranes show lower selectivities than the pure matrix (Φ = 0) and have higher permeances. Further investigations with lower adsorption agent content or smaller particles that create less voluminous voids can shift the selectivity to its optimum. Furthermore, for the design of a functioning membrane, the bridging between matrix and HTlcs needs to be ensured. Part 1 of this study showed evidence of those bridges forming; however, this process seems to be a challenge deserving optimization. This could be achieved with further manipulation of the boundary surfaces for faster polymerization via bridging oxygens between octahedral layers of the HTlc and silica. Combining this research with the recent efforts in organo–silica membranes can improve the performance of the matrix 10-fold, as the newest organo–silica membranes show better performance than the one used here [49]. In addition, the allocation of third-party bridging agents to help close the voids seems a possible approach [44].

## 5. Conclusions

This work offers a new design approach to fabricating membranes for high-temperature gas separation. LDH of hydrotalcite type was synthesized, delaminated, and made into a 200 nm thick membrane, which provides highly CO_2_-affinitive adsorption sites after calcination. As expected, the resulting pore size distribution favors unselective Knudsen diffusion. The microporous membrane, made of amorphous organo–silica, shows selective permeation behavior up to 150 °C. Combining both materials into a novel mixed-matrix membrane comprised of two inorganic materials proves to be possible. Although the functionality of the mixed organo–silica–HTlc membrane seems still to be temperature-limited for gas separation, these materials provide a large field for optimization. A combination of unselective Knudsen diffusion and selective surface diffusion explained the observed permeation behavior of the MMM. Incorporating the crystalline adsorption agent into the microporous structure is assumed to create voids at the interfaces that favor Knudsen diffusion. As a main objective for further research, the optimization of both pore size distribution of the gel matrix as well as the improvement of the connectivity between the gel bridging sites and octahedral layers will be identified.

This work establishes the foundation for membranes of this kind and fosters research for finding ways to overcome the bridging problems between both components.

## Figures and Tables

**Table 1:** Calculated single-gas, stand-alone, single-layer permeances; permeabilities; and respective ideal selectivities at 25 °C. Measured layer thickness derived from SEM images are 2.2 mm for α-Al_2_O_3_, 4 µm for γ-Al_2_O_3_, and 100 nm for Org-Sil, HymOS1, and HymOS2.

Single Layer	Permeance (10^−7^ mol (m^2^ s Pa)^−1^)		Permeability /10^−14^ mol (m s Pa)^−1^		Perm- Selectivity CO_2_/N_2_
	N_2_	CO_2_	N_2_	CO_2_	
α-Al_2_O_3_	4.5	3.6	99,000	79,200	0.8
γ-Al_2_O_3_	36.0	61.2	1440	2448	1.7
HTlc	14.7	12.3	29.5	24.6	0.8
Organo– silica	0.1	2.1	0.1	2.1	20.5
HymOS1	0.7	3.8	0.7	3.8	5.2
HymOS2	3.3	19.7	3.3	19.7	6.0

## Data Availability

The data that support the findings of this study are available from the corresponding author upon request.
